# On the spatial phase distribution of cutaneous low-frequency perfusion oscillations

**DOI:** 10.1038/s41598-022-09762-0

**Published:** 2022-04-09

**Authors:** Stefan Borik, Simon Lyra, Volker Perlitz, Micha Keller, Steffen Leonhardt, Vladimir Blazek

**Affiliations:** 1 Department of Electromagnetic and Biomedical Engineering, Faculty of Electrical Engineering and Information Technology, University of Zilina, Zilina, Slovakia; 2 Medical Information Technology (MedIT), Helmholtz-Institute for Biomedical Engineering, RWTH Aachen University, Aachen, Germany; 3 Simplana GmbH, Aachen, Germany; 4 Department of Psychiatry, Psychotherapy and Psychosomatics, Medical School, RWTH Aachen University, Aachen, Germany; 5 The Czech Institute of Informatics, Robotics and Cybernetics (CIIRC), Czech Technical University in Prague, Prague, Czech Republic

**Keywords:** Biomedical engineering, Cardiology, Health care, Optics and photonics

## Abstract

Distributed cutaneous tissue blood volume oscillations contain information on autonomic nervous system (ANS) regulation of cardiorespiratory activity as well as dominating thermoregulation. ANS associated with low-frequency oscillations can be quantified in terms of frequencies, amplitudes, and phase shifts. The relative order between these faculties may be disturbed by conditions colloquially termed ‘stress’. Photoplethysmography imaging, an optical non-invasive diagnostic technique provides information on cutaneous tissue perfusion in the temporal and spatial domains. Using the cold pressure test (CPT) in thirteen healthy volunteers as a well-studied experimental intervention, we present a method for evaluating phase shifts in low- and intermediate frequency bands in forehead cutaneous perfusion mapping. Phase shift changes were analysed in low- and intermediate frequency ranges from 0.05 Hz to 0.18 Hz. We observed that time waveforms increasingly desynchronised in various areas of the scanned area throughout measurements. An increase of IM band phase desynchronization observed throughout measurements was comparable in experimental and control group, suggesting a time effect possibly due to overshooting the optimal relaxation duration. CPT triggered an increase in the number of points phase-shifted to the reference that was specific to the low frequency range for phase-shift thresholds defined as π/4, 3π/8, and π/2 rad, respectively. Phase shifts in forehead blood oscillations may infer changes of vascular tone due to activity of various neural systems. We present an innovative method for the phase shift analysis of cutaneous tissue perfusion that appears promising to assess ANS change processes related to physical or psychological stress. More comprehensive studies are needed to further investigate the reliability and physiological significance of findings.

## Introduction

The skin is involved in multiple functions pivotal for the entire organism. Skin perfusion is thus a rich and easily available source of information on these functions, such as the activity of the autonomic nervous system (ANS), cardiorespiratory exercise as well as dominating thermoregulation^1,2^. Understandably, scientific zeal was sparked also at the prospect of obtaining a non-invasive measure of a clinically relevant system involved in serious mental and physical disorders. In that vein, studies of skin perfusion have a longstanding history. First published in 1937, photoplethysmography (PPG) has been demonstrated to be an undemanding and non-invasive technology for the study of the perfusion of superficial skin layers^3^. This approach has been advanced in current technologies to non-contact instruments used in cardiovascular diagnostics^4^.

For long skin perfusion studies focused mostly on understanding pulsatile PPG qualities, whereas the information on ANS processes in PPG signals remained insufficiently understood. Earliest references to proprietary oscillations in PPG signals as ‘psychomotor waves’^5^ had never been confirmed by objective correlates. The systematic analysis of frequencies and amplitudes inherent to PPG signals (or laser Doppler fluxmetry, LDF) has only as of late demonstrated that a mere 2.5% fraction of the total signal power originated in cardiovascular activity^6^. This is of interest to medical fields specializing on the non-invasive and non-contact cardiovascular diagnostics^4^.

Scientific study of ANS influence on cardiovascular-respiratory processes focused chiefly on a low and a high frequency band, LF and HF, resp. Those variance categories were, however, matter of scientific controversy with respect to the boundaries and physiological representations of the LF-band since it is considered to contain sympathetic as well as parasympathetic nervous traffic. While the HF-band is commonly agreed to represent vagal (parasympathetic) activity found in respiratory sinus arrhythmia (RSA), a state of weak coupling between cardiac and respiratory activity^7^, the validity of the entire approach is questionable if one of the frequency bands evades evidence^8–10^. This, unfortunately, impeded its clinical use to this day.

An approach which appears to overcome the dilemma on ANS-related frequency bands was introduced with findings on a 0.15 Hz rhythm or intermediate (IM) band. Wedged between the LF and the HF frequency band at 0.12 Hz to 0.18 Hz, this IM-band bridges the frequency boundaries of upper LF and lower HF band. A first description of the IM-band was given for skin LDF data by Smits et al. during hyperventilation^11^. Further studies showed the IM-band strongly associated with profound relaxation in skin of earlobe, forehead, and heart^12^. Controlled relaxation using self-control techniques such as the autogenic training (AT) exhibited during extended IM-band prevalence cardiorespiratory phase synchronizations (CRPS) at integer number ratios, so-called *n*:*m*—synchronizations^13^. In particular, these findings suggest a series of lower and upper harmonics of the primary IM-band frequencies covering essential LF- and HF-frequencies. This suspends the role of distinct frequency bands as it emphasizes instead the relevance of ratios of interacting frequencies. Comparison of human multiple physiological time series with canine experiments showed widely identical frequency characteristics in spite of scaling differences which suggested identical origin of the IM-band in unspecific reticular neurons in the common brainstem system^13^. This is of note since Traube speculated already in 1865 on phasical ‘irradiations’ of medullary respiratory centers on brainstem nuclei controlling heart rate^14^.

Low-frequency oscillations exhibit spatially variable amplitudes, which depend on the site of the PPG probe. Therefore, the photoplethysmography imaging (PPGI) method, first described in^15^, is ideal for the spatial analysis of skin perfusion dynamics, which explains increased interest of this method^16^. PPGI enables spatial–temporal analysis of the distribution of perfusion oscillations using a camera system^17^ combined with an external light source^18^, or use of ambient illumination only^19^.

According to^20^, the PPGI method can be used to differentiate low-frequency oscillations in healthy and damaged tissues. This research group demonstrated also that slow oscillations were not phase synchronized.

In spite of open questions concerning frequencies and amplitudes in PPG-signals, the phase-shift is an essential yet still “*under-explored*” parameter, which therefore mandates systematic study^19^. This approach was employed by^21^, using a technique published first by^22^ to monitor the relative phase of perfusion changes associated with the cardiac activity in migraine patients.

This research group^23^ validated their findings with a novel model of light-tissue interaction when using a reflective and non-contact PPG, i.e. PPGI technique. The authors reported that the transmural pressure of the larger arteries apparently affected locations in cutaneous areas where HR-related blood volume changes oscillate at opposite phase to the reference signal acquired from another location on the skin. Thus, depending on the wavelength used HR-related changes in the PPG signal detected with PPGI may be induced indirectly by oscillations of larger arteries located even in deeper subcutaneous tissue structures^24^. In contrast^25^, another view on light-tissue interaction for monitoring the entire experiment is given by videocapillaroscopy.

The current communication describes a novel method for mapping the phase shift of perfusion fluctuations in cutaneous tissues. In contrast to mapping cardiac activity, we focused on -frequency oscillations in the range between 0.05 Hz and 0.18 Hz. We divided this frequency band into low-frequency (LF) sections and intermediate (IM) sections with boundaries according to Pfurtscheller (_Pf)^26^ and Keller (_Ke)^27^. Thus, we studied four bands: LF_Pf (0.05 Hz – 0.10 Hz), IM_Pf (0.10 Hz – 0.15 Hz), and LF_Ke (0.05 Hz – 0.12 Hz), and IM_Ke (0.12 Hz – 0.18 Hz). To stimulate ANS responses, we used the well-studied cold pressure test in young healthy individuals to induce changes in the spatial distribution of the phase shift relative to the frontal region's central reference. We chose the correlation between phase synchronization of slow perfusion rhythms and possible changes in ANS regulation caused by stress as an outcome parameter. To the best of our knowledge, this approach has never been probed previously.

## Materials and methods

### Experimental protocol

**Figure 1:**
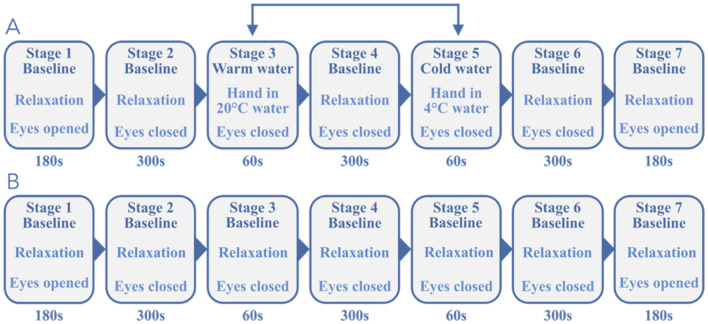
(**A**) Stages of the experimental protocol in the experimental group (*N* = 13); with stages 3 and 5 being applied in reversed order in 3 recordings to randomize intervention. (**B**) Stages of the control group (*N* = 3) with all stages using identical conditions.

A total of 16 subjects aged (27.0 ± 2.2) years participated in the study. The right- or left-handed individuals were non-smokers and had no acute physical or mental illness, as was tested using the German version of the patient health questionnaire (PHQ-D^28^;). To avoid falsifying measurement outcomes, subjects were asked to refrain from consuming anything containing caffeine on the day of the experiment. We used a between-subject blocked protocol with seven stages in a supine position to allow internal and external control of ANS stimulation. In 13 subjects, the following sequence was applied: (1) Eyes Open (EO), (2) Eyes closed (EC), (3) EC + cold pressor/ambient water test (CPT, AWT), (4) EC, (5) EC + cold pressor/ambient water test, (6) EC, (7) EO. Stages 1 and 7 lasted 180 s, and stages 2, 4, and 6 lasted 300 s. Stages 3 + 5 lasted 60 s and were applied in randomized order. Stage 3 or 5, CPT or AWT, was performed by immersion of the subjects’ non-dominant hand (up to the carpus) in water of either 4 °C or water of ambient temperature (AWT) of 20 °C for 60 s. In another group of 3 subjects, conditions of stage 2 (EC) were applied also in stages 3 and 5 to serve as a time control group. Breathing was not controlled to avoid any unintended stress by external control. Furthermore, the head was not fixed to minimize motion artifacts since this could also induce stress. The hand was dried afterwards using a towel placed on the patient's abdomen. Stage 6 (EC) and stage 7 (EO) finalized the recording. An overview of all stages is illustrated in Fig. 1A,B. The whole experiment lasted 23 min in total and was well tolerated by all participants. The study protocol was approved by the ethical committee of the University Hospital at RWTH Aachen University (Ref. No. EK 219-21), informed consent was obtained from all subjects prior to the study, and all methods were performed in accordance with the study protocol and with the Declaration of Helsinki.

### Measurement setup

The measurement setup used a high-performance complementary metal-oxide-semiconductor (CMOS) monochrome camera of type Grasshopper 3 GS3-U3-23S6 M-C (FLIR Systems, USA), with a fixed focal length lens of type Fujinon CF12.5HA-1 (Fujifilm Holdings, Japan) mounted to the camera. The recordings were performed with a spatial resolution of 1920 × 1200 pixels (12 bit per pixel) at 30 fps. Since ambient illumination was necessary for the measurements, four organic LED (OLED) panels of type Keuka warmwhite 24 V (OLEDWorks, Germany) were mounted next to the camera. The advantages of using OLEDs instead of LEDs for camera-based vital sign extraction were described in^29^. A similar setup using one OLED to record simulated PPGI signals from an optoelectronic skin perfusion phantom was described earlier^17^.

**Figure 2:**
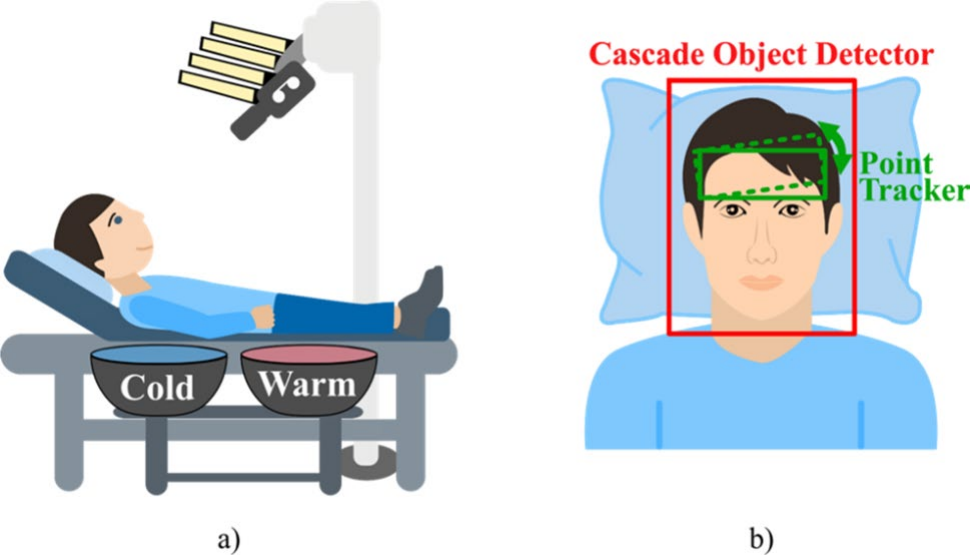
(**a**) Measurement setup. (**b**) Example of the field of view with an applied face detector and forehead tracking.

In this study, a comfortable patient bench was placed beneath the camera setup to locate the participants in a supine position. The angle of the patient’s head to the camera was adjusted using a pillow. This ensured optimal conditions for continuous measurement of facial skin perfusion. Two bowls, one with iced water (approx. 4 °C), another one with water at ambient temperature (approx. 20 °C) were placed within the reach of the subject to allow low-movement immersion of the hand. The measurement setup is shown in Fig. 2a. Figure 2b illustrates the view of the camera and indicates the object detector and the point tracker used for automated region extraction explained below.

### Data processing

**Figure 3:**
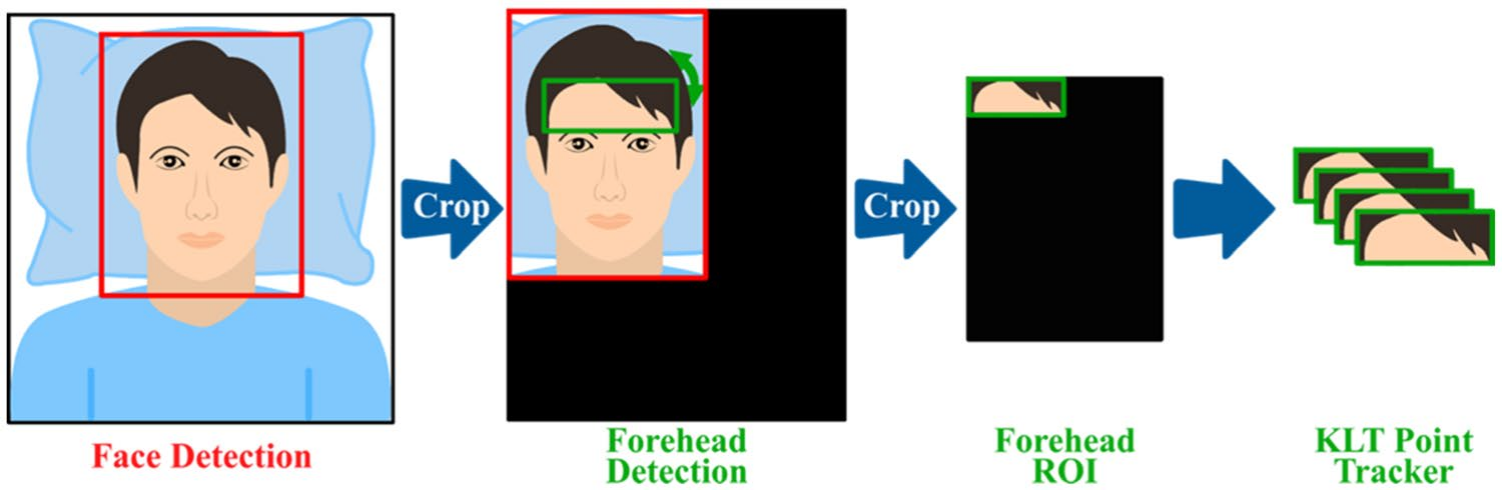
Face and forehead ROI tracking.

As the first step, raw data were prepared to evaluate the spatial distribution of the PPGI signal phase by determining the desired area of interest; here, we chose the subjects’ forehead. The following precautions had to be taken prior to initiating an extended recording (23 min), as was done in this case. First, the subject's lateral head movements have to be controlled since they can cause signal artifacts that are amplified given the small kernel size used to extract temporal PPGI signals from the monitored image matrix. We decided to track the selected area of interest, which has been previously used^30–33^. Second, since this tracking algorithm can result in image jittering^34^, we used double tracking by combining the Viola-Jones algorithm^35^ with the point detector method^36–38^. Here, the face detector automatically detected and extracted the face area as a bounding box, which was then magnified by 10% to cover the entire face. Third, the image was cropped and inserted into the auxiliary image layer (filled with zeros), ensuring the exact image size for subsequent processing and tracking using the KLT point tracker. At this stage, a rectangle containing a region of interest (ROI) on the subject's forehead was manually selected. Fourth, the KLT point tracker was initialised to track and extract the selected ROI using minimal eigenvalue features^36^. In the case of angling the subject’s face off the horizontal plane, detected ROIs were automatically reprojected into the image's original horizontal plane perpendicular to its symmetry axis. According to its upper edge, the process began by calculating the bounding box's angle of inclination. The image was then rotated back and inserted into another unified image layer and saved in *.pgm format. Prior to data analysis, pre-processing of image data was finalized by resizing all images to the selected reference, the first image in the sequence of measured data. This compromise was due to the limitations of tracking and the fact that various sized ROIs were detected as subjects moved. To minimize mixing of individual pixels, the nearest extrapolation method was chosen. The modified image was then saved to the hard disk. Figure 3 illustrates the process of face and forehead ROI tracking.

**Figure 4:**
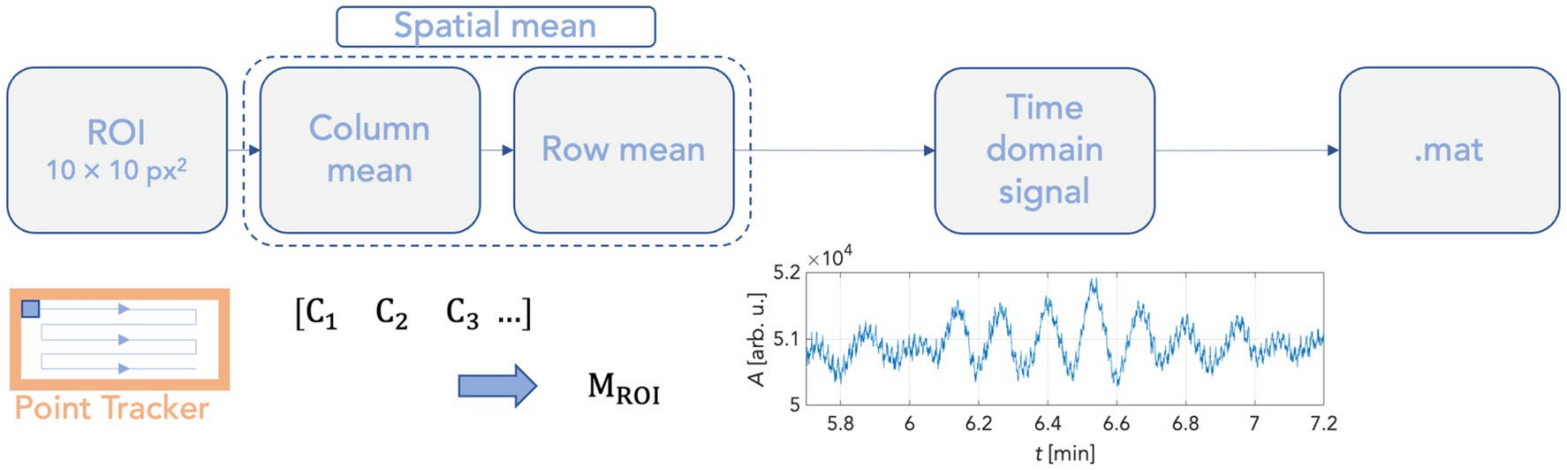
Process of obtaining PPG signals in time domain.

**Figure 5:**
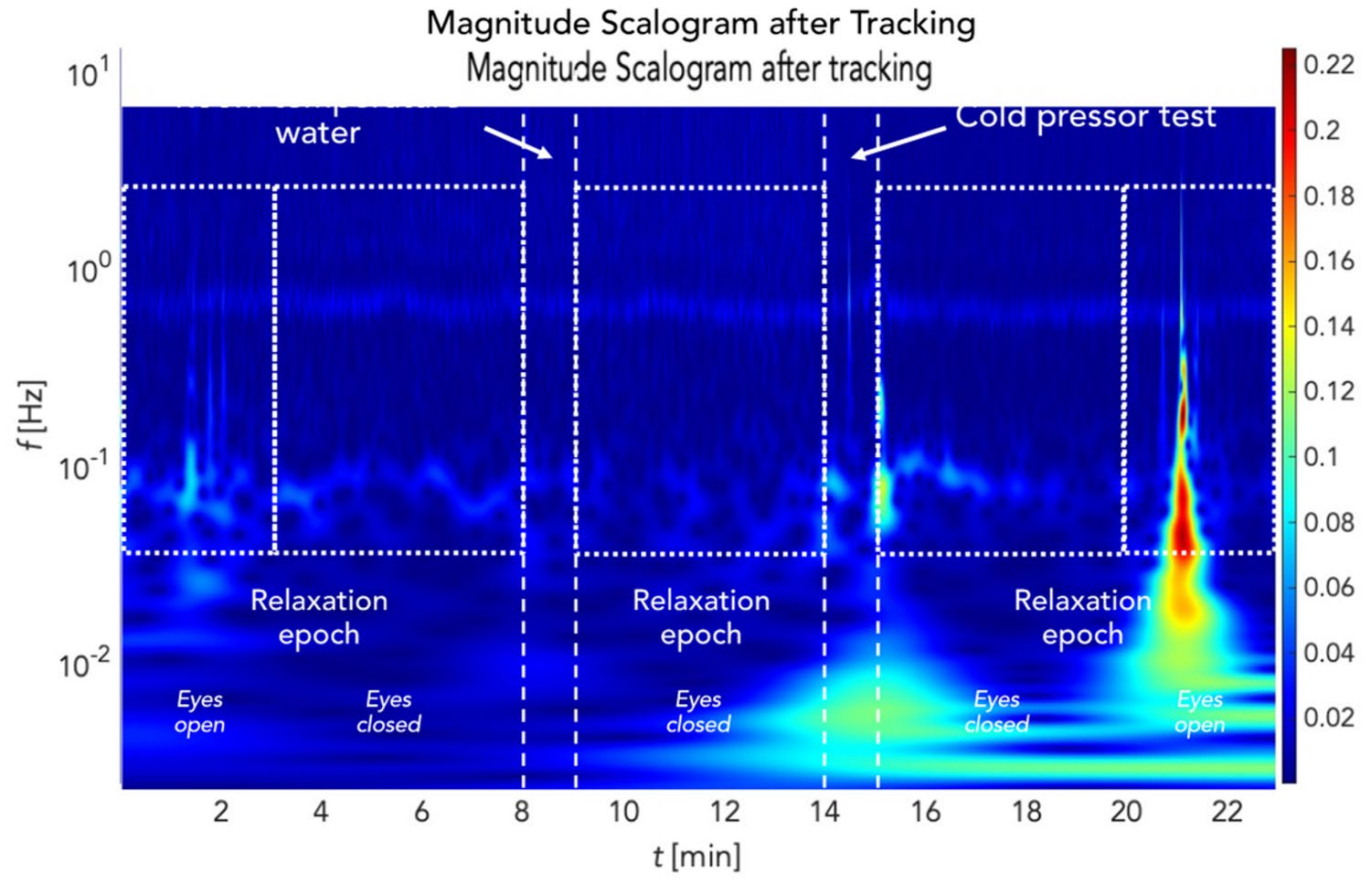
Continuous wavelet scalogram after tracking.

**Figure 6:**
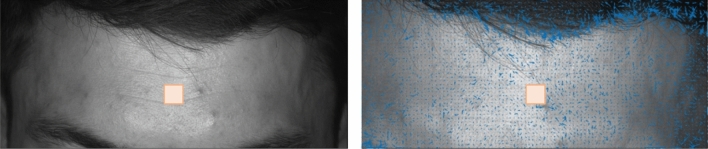
Mimics and light change comparison (at the start—frame 1, in the middle of cold pressure test—frame 25,989). Blue colour depicts motion estimated by optical flow.

Following pre-processing signals from sufficiently stable ROIs, the time courses of the PPGI signals were extracted by analysing each recorded frame. Time waveform extraction was initiated by selecting a 10 × 10 px^2^ kernel moving without overlap in the selected forehead region (Fig. 4). The spatial mean was then computed for each kernel position of the processed frame. Thus, a three-dimensional matrix of time-domain signals was obtained. Two dimensions encoded the extracted signal's spatial location. The third dimension was a time vector at a length equal to the number of frames recorded (41,400 samples = 23 min). Figure 5 depicts a wavelet scalogram of the unfiltered signal from a 10 × 10 px^2^ region close to the subject's forehead showing perfusion rhythms in the frequency domain. This image still contained primarily artifacts due to facial expressions and changes in the geometry of the tracking area, which persisted despite image tracking (Fig. 6). Suppression of these artifacts was achieved using the stationary wavelet transform (SWT)^39–41^. Prior to SWT denoising, the signal was downsampled to the sampling frequency of 5 Hz.

This decomposed the signal using Daubechies – db5 wavelets—to the eight levels processing each detail level separately using a moving window to compute the median and standard deviation. This helped to obtain the envelope of a particular wavelet decomposition detail. Subsequently, all values below this envelope were adaptively filtered separately using soft thresholding for each decomposition level. Finally, the signal was reconstructed using inverse SWT. The purpose of this entire process was not to eliminate noise but to determine it. The estimated noise was then subtracted from the original signal, eliminating other undesired artifacts, and preparing the signal for further processing and analysis.

After removing these artifacts, defined frequency bands were extracted using finite response (FIR) band-pass filters using the following parameter settings: low frequency (LF)_Pf band (cut-off frequencies 0.05 Hz – 0.10 Hz); intermediate (IM)_Pf band (cut-off frequencies 0.10 Hz – 0.15 Hz); LF_Ke band (cut-off frequencies 0.05 Hz – 0.12 Hz); IM_Pf band (cut-off frequencies 0.12 Hz – 0.18 Hz).

This signal processing focused on observation of ANS regulation in those frequency ranges, which have been recently demonstrated to be distinct processes^27^. These frequency bands may contain respiration-induced perfusion changes^42^. However, there is evidence that while IM band activity and respiration are closely related processes, they are yet distinct^10,43^. This is supported by other authors reporting LF changes associated with waves formerly referred to as Mayer-Traube-Hering oscillations being only slightly affected by respiration^44,45^.

**Figure 7:**
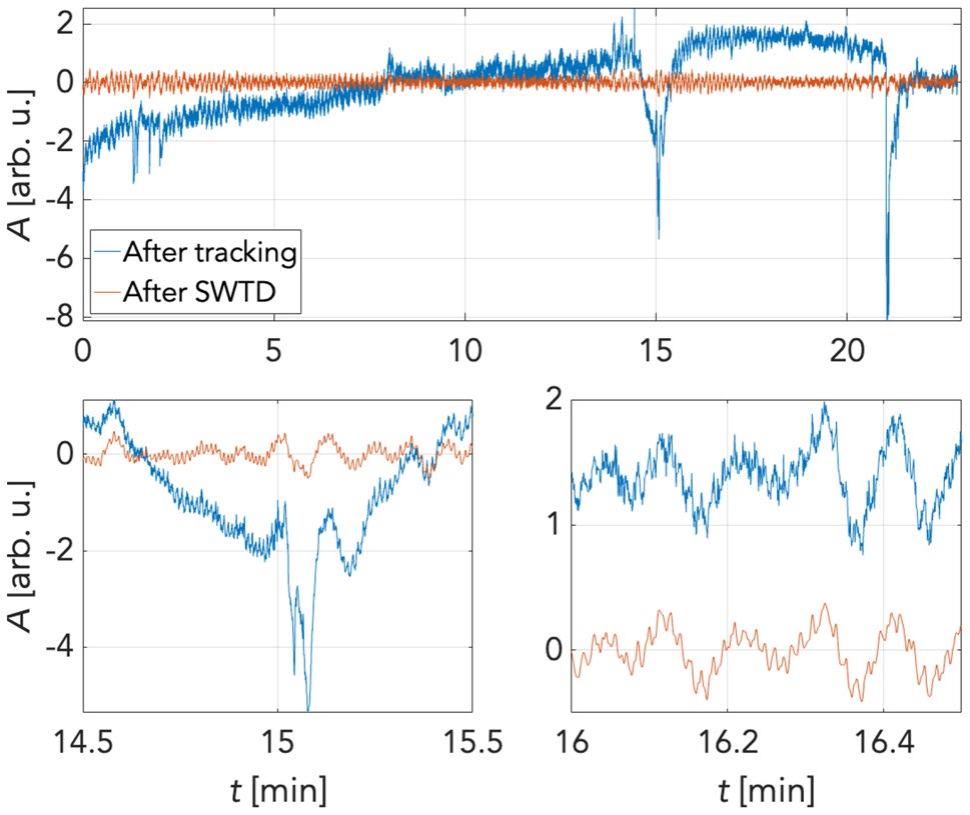
Signal before and after stationary wavelet transform denoising.

**Figure 8:**
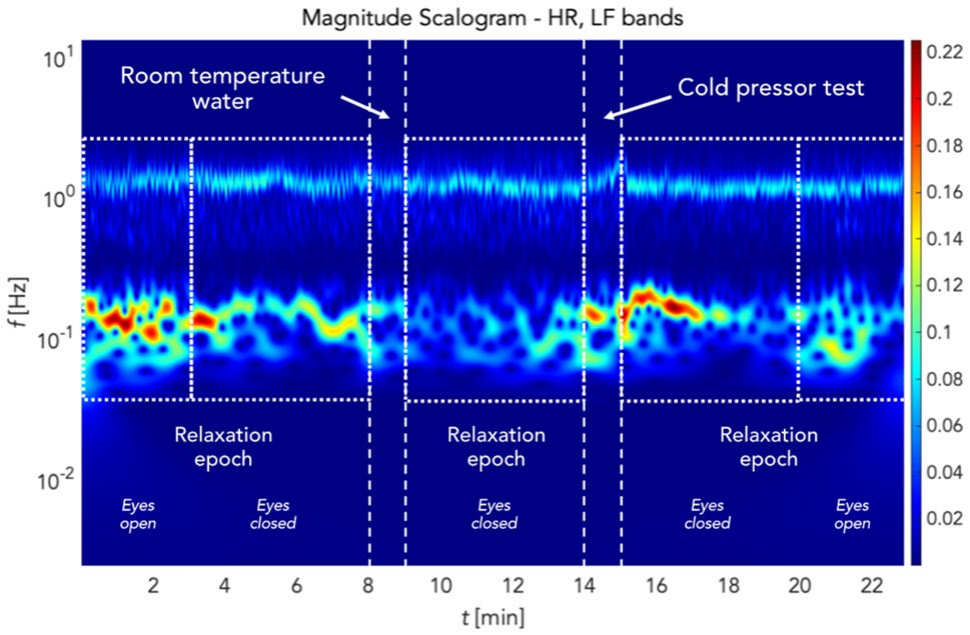
Filtered scalogram showing heart rate (HR) and low frequency (LF) bands and experimental stages.

The effects of filtering using stationary wavelet transform denoising (SWTD) can be seen in Fig. 7. The blue curve represents the time-domain signal following tracking, while the red curve corresponds to the signal following denoising using the SWTD algorithm. As illustrated in this figure, filtering helped suppress artifacts caused by the subject's facial expressions, especially the forehead wrinkling visible in Fig. 6. Furthermore, this figure shows PPG signals from which ROIs were extracted. Along with suppressed artifacts, it is possible to see a smoothed PPG curve and the oscillations corresponding to slow changes in cutaneous tissue perfusion. After removing noise and artifacts, the signal reconstruction process included only wavelet levels whose frequency corresponded with LF oscillations as defined above. Simultaneously, a frequency band for the heart rhythm up to approx. 5 Hz was included to observe possible higher harmonic components corresponding to the dicrotic notch in the PPG signal. Figure 7 shows a signal filtered this way, which exhibits artifact suppression even in the frequency domain (Fig. 8).

Further filtering of spectral components helped avoid inaccuracies in determining the phases of LF oscillations, which might possibly be affected by other physiological oscillators, such as cardiac activity or respiration. The Hilbert transform was chosen to provide information on the instantaneous phase of the signal as this is a computationally undemanding yet robust and well studied approach. The phase is determined separately for each time waveform of the PPGI matrix. Applying the Hilbert transform yielded an analytical signal:

1 $$x(t) = x_{\text{r}} + {\text{j}}x_{{\text{i}}} ,$$

where *x*_r_ represents the original signal and *x*_i_ is its imaginary part shifted by 90°.

The information on the instantaneous phase is:

2 $$\phi (t) = \text{atan} \frac{x_{\text{i}}}{x_{\text{r}}},$$

where *ϕ*(*t*) has the form of a sawtooth signal carrying information on changes of the phase of the signal within a given cycle. This allowed calculation of the instantaneous phase of each time course of the PPGI matrix, creating a three-dimensional instantaneous phase matrix carrying spatial-temporal information on phase distribution in the forehead region and changes over time.

**Figure 9:**
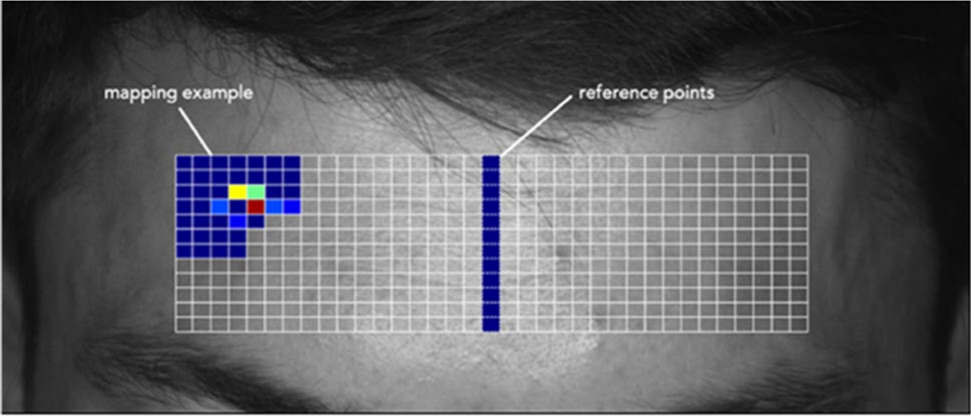
Example of map creation. Reference points in the matrix are placed in the middle of each row.

Selection of a suitable reference signal was essential to create a planar map of phase changes. Here, the axis of symmetry was determined using the central vertical line of the forehead region. This reference signal was always central element for each row of the phase matrix, which consisted here of a uniform size of 12 rows and 35 columns (Fig. 9).

The algorithm used to create this map started by computing the phase difference between the reference point and all other points (column positions) in any given row. This allowed to estimate the relative phase shift across all phase matrix points. Thus, a matrix of dimensions 12 × 35 was obtained, with the points on the symmetry axis naturally equal to zero radians.

The phase shift between the reference and the corresponding matrix point was calculated as the absolute value of their instantaneous phase differences within a specified time window.

We chose a 10∙*F*_s_ window size to avoid sudden and undesirable phase changes caused, for example, by insufficient artifact suppression. This window of 3000 samples (100 s) moved acrosss the entire signal, calculating the median ${\phi }_{\mathrm{med}, r,c}\left(t\right)$ of the difference between the absolute phase value of the reference signal ${\phi }_{\mathrm{ref}, r}\left(t\right)$ and the signal of the corresponding point ${\phi }_{r,c}\left(t\right)$ of the phase shift matrix:

3 $$\phi_{{\text{med,}r,c}} \left( {{t}} \right) = {|}\beta {|} = \left| {\phi_{{\text{ref,}r}} \left( {{t}} \right) - \phi_{r,c} \left( {{t}} \right)} \right|{,}$$

where *r* and *c* represent rows and columns of the phase matrix, respectively.

**Figure 10:**
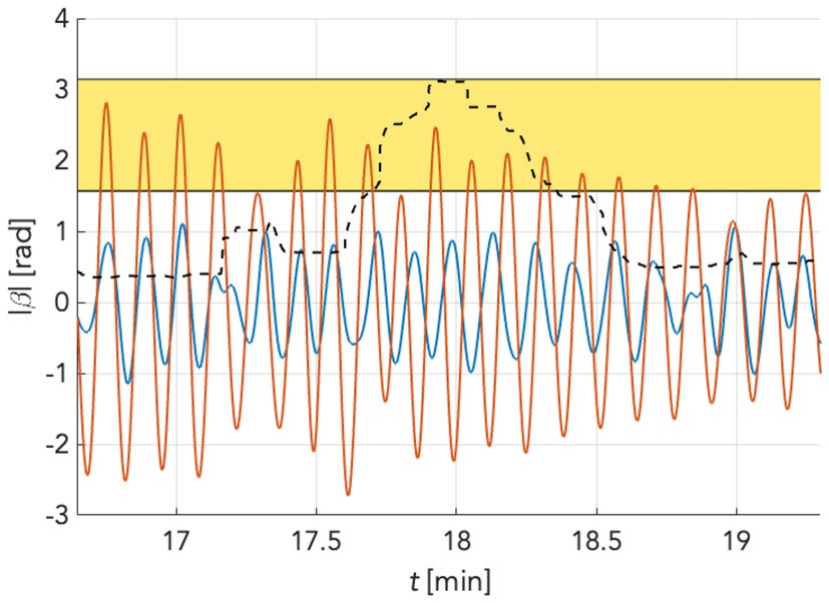
Detection of desynchronization based on the Hilbert transform. Blue and red signals represent ROIs from different parts of the forehead. There are low-frequency oscillations obtained from the signal by SWTD, similar to initial denoising. The dashed black line is the instantaneous phase calculated in the moving window with a length of 10 s. The area marked yellow corresponds to the desynchronization threshold used for further analysis.

An example of this process is shown in Fig. 10, depicting changes in the phase synchronization between the reference point and a selected point of the phase matrix corresponding to the forehead region of the subject under investigation.

To create a temporal sequence of phase maps and visualize the actual phase shift changes and their spatial distribution, a moving window of 60 s was used to calculate the average phase shift value $\overline{\beta }$ for each point of the phase matrix. Since averaging failed to correct averaging of circular quantities, a method incorporating its vector properties was used instead:

4 $$\overline{\beta } = {\text{atan2}}\left( {\mathop \sum \limits_{{{n = 1}}}^{{{W}}} {\text{sin}}\beta_{{{n}}} {,}\mathop \sum \limits_{{{n = 1}}}^{{{W}}} {\text{cos}}\beta_{{\text{n}}} } \right){,}\;{\text{or}}\;{\text{in}}\;{\text{complex}}\;{\text{numbers }}\;\;\overline{\beta } = {\text{arg}}\left( {\mathop \sum \limits_{{{n = 1}}}^{{{W}}} {\text{ e}}^{{{\text{j}}\beta_{{{n}}} }} } \right){,}$$

where ${\beta }_{n}$ is the relative phase of selected points of the phase matrix, *n* corresponds to the frame number and *W* corresponds to the moving averaging window length.

This logic was followed when selecting phase thresholds to analyse the synchronisation of spatial changes in perfusion. First, we wanted to use intervals that would not overlap, as is usually the case, for example, when selecting frequency bands in amplitude analysis of signals. However, instead of this approach, we used phase thresholds and thus intervals with overlaps, i.e., from the left, each interval is bounded by a π value. This choice is supported by the assumption that the relative temporal position between the reference and the observed signal is tracked at a selected point in a matrix containing information about the spatial distribution of perfusion in the low-frequency domain when working with phase shifts. Furthermore, we assume that the perfusion signals (due to their nature) are not perfectly aligned, and even in the case of resting state, some initial phase shift is present between them (here considered as background). By gradually narrowing the interval towards the π value, the responses to the intervention (CPT or AWT) become apparent and are differentiable from the background.

**Figure 11:**
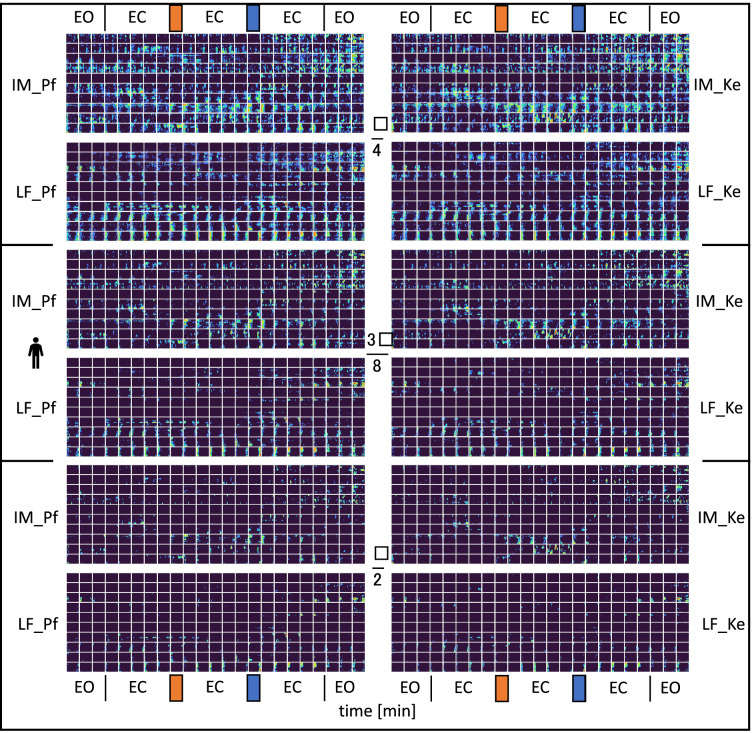
Spatial phase shift distribution matrices indexing desynchronization for low and intermediate frequency (LF, and IM, resp.) bands according to boundaries used by Pfurtscheller (Pf, left column) or Keller (Ke, right column) for individual participants (horizontal lines of squares) of the main group (n = 10). EO = eyes open, EC = eyes closed; orange bar at top: cold water test, blue bar at top: ambient water test. From top to bottom, colour coded points display thresholds for relative phase shift intervals from π/4 rad to π rad, 3π/8 rad to π rad, and π/2 rad to π rad: dark blue < blue < green < yellow. Red indexing counterphase with the reference. Plots show only minute differences between Pfurtscheller and Keller frequency definitions.

Thus, to map spatiotemporal variation of phase, the values with an average phase shift less than π/4 were marked blue, which corresponded to zero, and phase shift values in the range of π/4 to π were colour coded from green to red, indicating counterphase to the reference. This approach was also used for colour coding of the 3π/8-π and π/2-π intervals. The spatiotemporal variation in reciprocal phase shifts is illustrated in Fig. 11, which depicts minute-by-minute changes. The y-axis included information on the measured subjects. Another technique of representing the relative phase shifts was to encode them following their time course. This was achieved by counting the number of phase matrix points with a relative phase shift to the reference exceeding a predefined threshold in a given row. Here, three different threshold values (*thrs*) π/4, 3π/8 and π/2 rad were defined. Subsequently, the ratio of phase matrix points exceeding the pre-set threshold to the total number of phase matrix points was calculated for each frame and, thus, for the associated phase matrix. This process is mathematically described by equation:

5 $${{R}}_{{\phi > {{thrs}}}} \left( {{t}} \right) = \frac{{{{N}}_{{\phi > {{thrs}}}} }}{{{N}}}{,}\;{{thrs}} = \left\{ {\frac{\pi }{{4}}{, }\frac{{{3}\pi }}{{8}}{,}\frac{\pi }{{2}}} \right\}{,}$$

where *N* = *r* × *c* is the total number of phase matrix points and ${N}_{\phi >thrs}$ is the number of points exceeding the predefined threshold.

To quantify relative phase changes within each group and phase shift threshold value (π/4, 3π/8 and π/2 rad), separate 2 (LF_Pf, IM_Pf and LF_Ke, IM_Ke) × 7 (Repetition) repeated measures analyses of variance (ANOVAs) of relative phase desynchronization were computed. The main experimental and random group were analysed together after adapting the order of AWT and CPT as well as subsequent EC stages of the random group to match the order of the main group. Despite lack of power due to a small sample size, the control group was also analysed using a repeated measures ANOVA. A *p*-value of < 0.05 was considered significant. Post-hoc paired *t*-tests were computed for (1) the contrast stage 1 versus 7 to test whether our experimental conditions were followed by an increase in phase desynchronization, and (2) contrasting AWT and CPT (stage 3 vs 5) to test differences in phase desynchronization. These post-hoc tests were conducted separately for phase shifts, groups as well as frequency band definitions. Bonferroni corrections were applied for each frequency band definition pair (LF_Pf/IM_Pf and LF_Ke/IM_Ke) and separately for each phase shift, resulting in an α = 0.05/4 = 0.0125.

## Results

**Figure 12:**
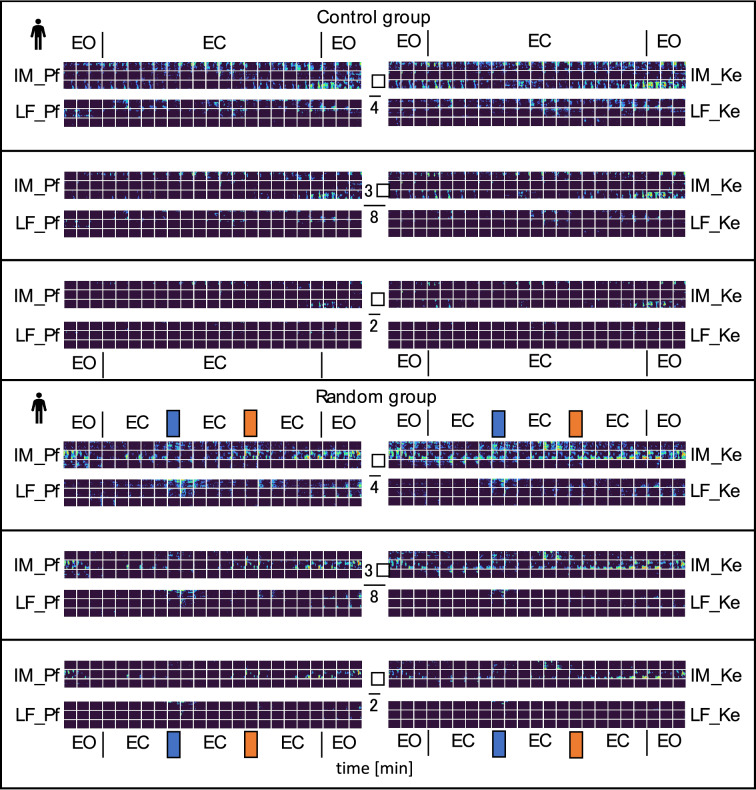
Spatial phase shift distribution matrices indexing desynchronization for low and intermediate frequency (LF, and IM, resp.) bands according to boundaries used by Pfurtscheller (Pf, left column) or Keller (Ke, right column) for individual participants (horizontal lines of squares) control and random groups. EO = eyes open, EC = eyes closed; orange bar at top: cold water test, blue bar at top: ambient water test. From top to bottom, colour coded points display thresholds for relative phase shift intervals from π/4 rad to π rad, 3π/8 rad to π rad, and π/2 rad to π rad: dark blue < blue < green < yellow. Red indexing counterphase with the reference.

The matrices of spatial phase shift distribution indexing desynchronization for low and intermediate frequency are shown for the experimental (main) group in Fig. 11 and for control and random groups in Fig. 12, resp. (results based on Eqs. (3) and (4)). These plots exhibit various patterns for phase shift threshold ranges, frequency bands, and experimental time. Pattern differences for Pf and Ke boundary definitions appear rather insignificant. Furthermore, phase shift distributions were plotted for π/4, 3π/8 and π/2 thresholds, and they were more prominent in either IM band as in LF bands. Thus, desynchronization was strongest in π/4 in the IM band. Conversely, there was almost complete synchronization with reference for π/2 in the LF band.

To quantify individual findings, using Eq. (5) we computed the median (red graphs) for phase shift changes (desynchronization compared to reference) for π/4, 3π/8, and π/2 rad thresholds in the main and the random group combined, and for the time control group. This was computed separately for LF and IM frequency bands at varying boundary definitions according to Pfurtscheller (Pf) and Keller (Ke). The increase in phase desynchronization can be interpreted as an increase in the number of matrix points above the predefined threshold, with a value of 1 equal to 100% phase shifted points compared to reference.

**Figure 13:**
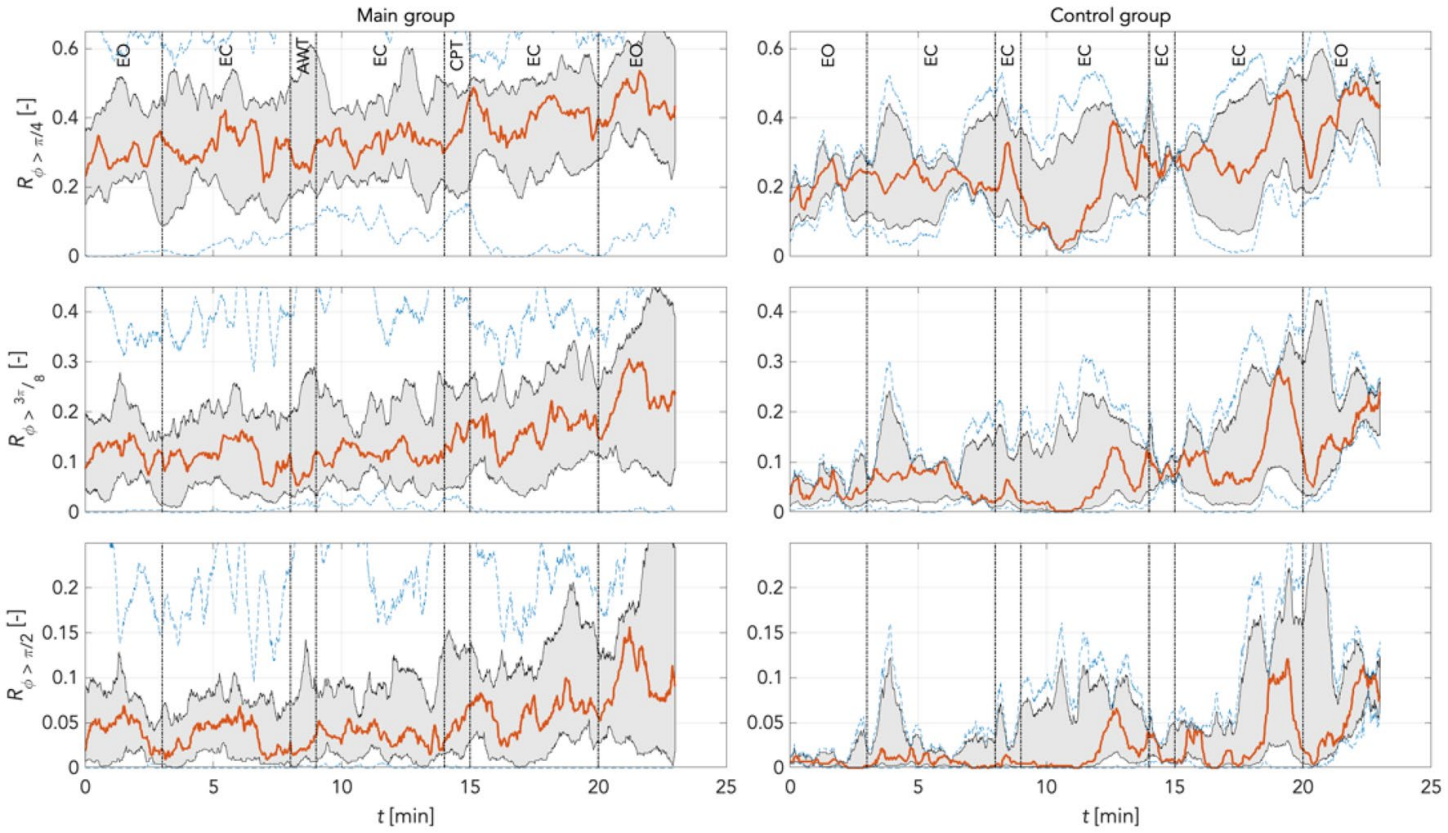
Phase desynchronization for different thresholds for main and control groups in the IM_Pf band (0.1 Hz – 0.15 Hz) *including* 0.1 Hz activity. There were only few oscillations in the main group. The increases in phase desynchronization due to CPT were of magnitudes comparable to other non-experimental oscillations. In controls, few prominent spontaneous oscillations. In both groups, phase desynchronization increased over time. Levels of phase desynchronization were π/4 > 3π/8 > π/2 rad in both groups. Red: median curve, black vertical lines: stages of the experiment, grey area bounds: 25^th^ and 75^th^ percentiles; blue dashed lines: min and max values.

**Figure 14:**
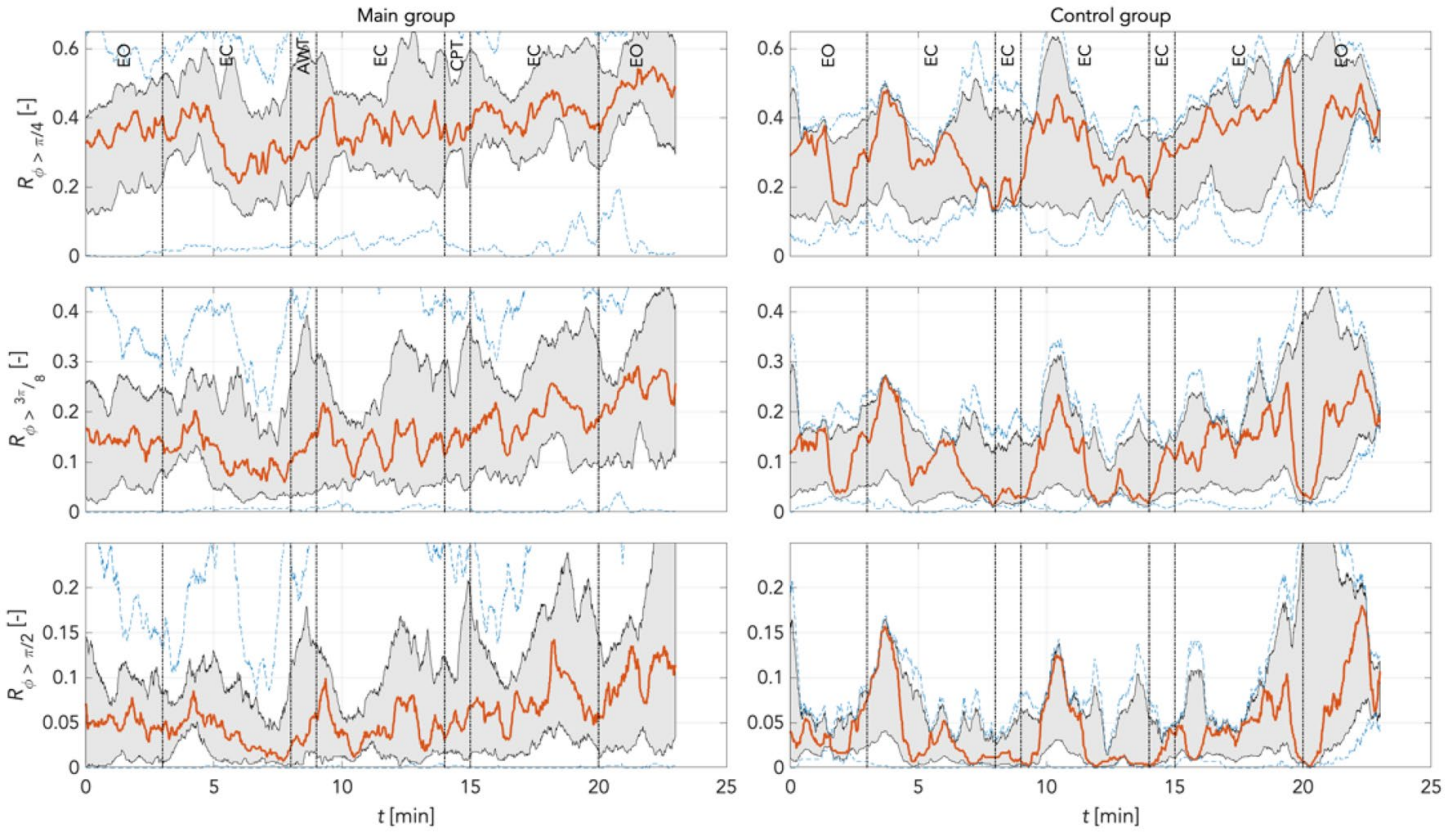
Phase desynchronization for different thresholds for main and control groups in the IM_Ke band (0.12 Hz – 0.18 Hz) *excluding* 0.1 Hz activity. In the main group, there were hardly any obvious responses to CPT. This group showed also only a slight increase of phase desynchronizations over time. Of note, the control group exhibits for all phase thresholds distinct peaks in phase desynchronizations of approx. 100 s length at minute 4 and 11. Phase desynchronizations increased distinctly at the end of the recordings in 3π/8 and π/2 thresholds. Red: median curve, black vertical lines: stages of the experiment, grey area bounds: 25^th^ and 75^th^ percentiles; blue dashed lines: min and max values.

Figures 13 and 14 shows the time courses of phase desynchronizations for the main and control groups for frequency boundaries defined as IM_Pf band (0.10 Hz – 0.15 Hz) and IM_Ke (0.12 Hz – 0.18 Hz), respectively. Two patterns are obvious in both groups and both frequency definitions. Firstly, the levels of phase desynchronizations decreased for thresholds with π/4 > 3π/8 > π/2 rad. In the IM_Pf band the smallest phase window (π/2 rad) underlines the upward trend throughout measurements pointed out by a more specific response. Secondly, phase desynchronizations increased over time for all three thresholds and both groups. However, there were fewer prominent oscillations in the main group during the entire experimental time course than in control. In the main group, phase desynchronization due to CPT were of magnitudes comparable with non-experimentally induced fluctuations in all thresholds. In controls, there were prominent spontaneous oscillations. Opening the eyes (stage 7) was followed by a transient increase in phase desynchronization in all thresholds. Here, the increase in phase desynchronization appeared to be strongest as of the 17 min. This may have been due to the length of the relaxation causing some discomfort for participants.

**Figure 15:**
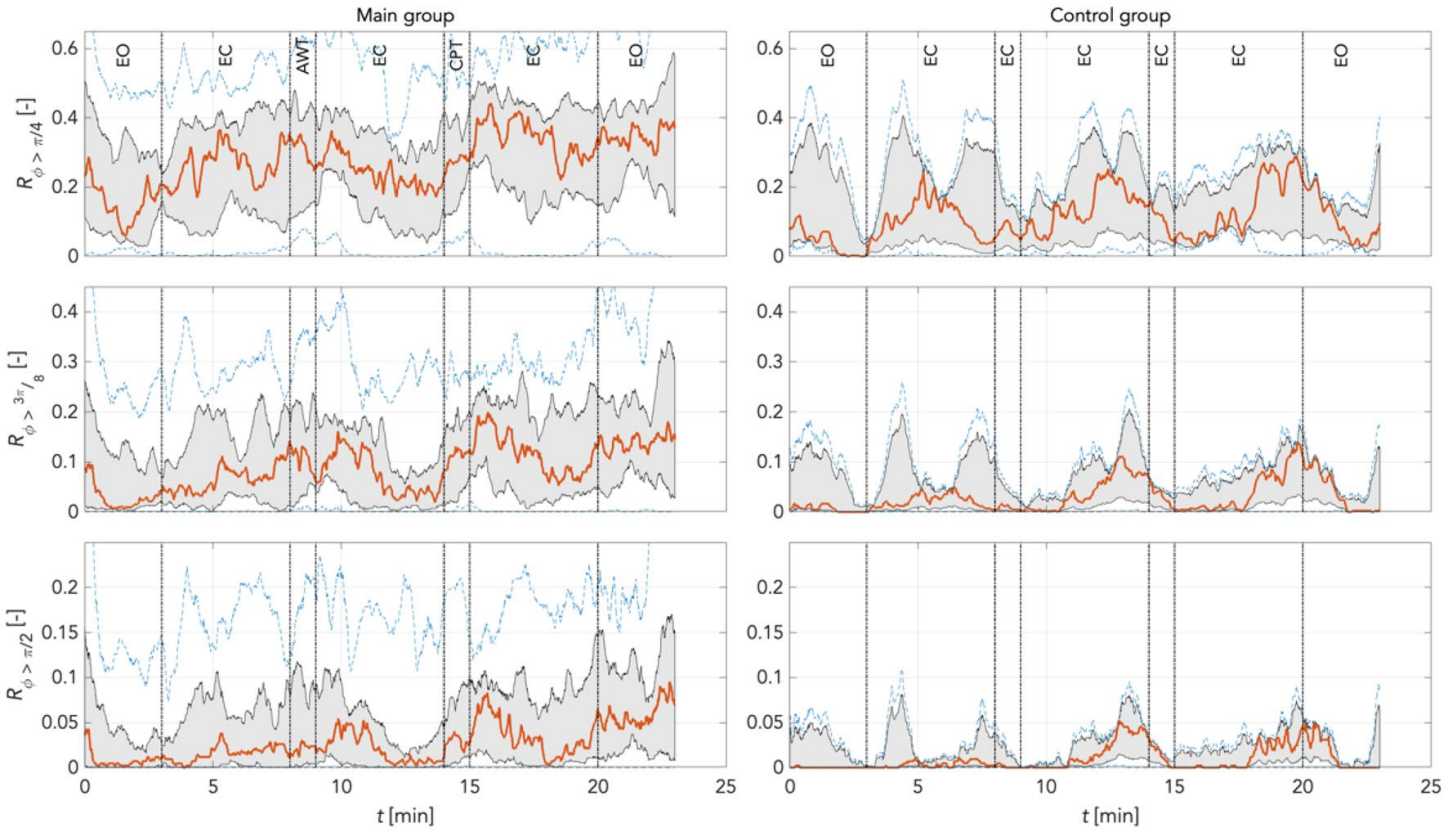
Phase desynchronization for different thresholds for main and control groups in the LF_Pf band (0.05 Hz – 0.1 Hz) *possibly curtailing* 0.1 Hz activity. In the main group, few pronounced oscillations; the level of phase desynchronization decreased with each threshold. Comparable course of phase desynchronizations in all thresholds for CPT: stable course during CPT, rapid increases, slow decreases for about 3 min. Few differently pronounced oscillations of shorter frequency and amplitude in all 3 thresholds. EO (stage 7) accompanied by hardly any changes in phase desynchronization. Stable conditions in the control group: distinct oscillations approx. every 7 min, most pronounced at π/4 > 3π/8 > π/2 rad. No evidence of increases in phase desynchronization with time. Red: median curve, black vertical lines: stages of the experiment, grey area bounds: 25^th^ and 75^th^ percentiles; blue dashed lines: min and max values.

Figure 15 shows the time courses of the phase desynchronizations for the main and the control groups for frequency boundaries defined as LF_Pf frequency band (0.05 Hz – 0.10 Hz). In the main group, there were still few pronounced fluctuations. Here, too, the level of phase desynchronization decreased with each threshold. In all thresholds, a comparable course was found in CPT: stable conditions during CPT, then rapid increases in phase desynchronization, then continuous decreases in phase desynchronization for 3 min. Occasionally in all 3 thresholds differently pronounced oscillations of shorter frequency and amplitude appeared. Opening the eyes was accompanied by hardly any changes in phase desynchronization. The control group showed very stable conditions. There were distinct oscillations about every 7 min, most pronounced at π/4 > 3π/8 > π/2 rad. There was no evidence of increases in phase desynchronization with time.

**Figure 16:**
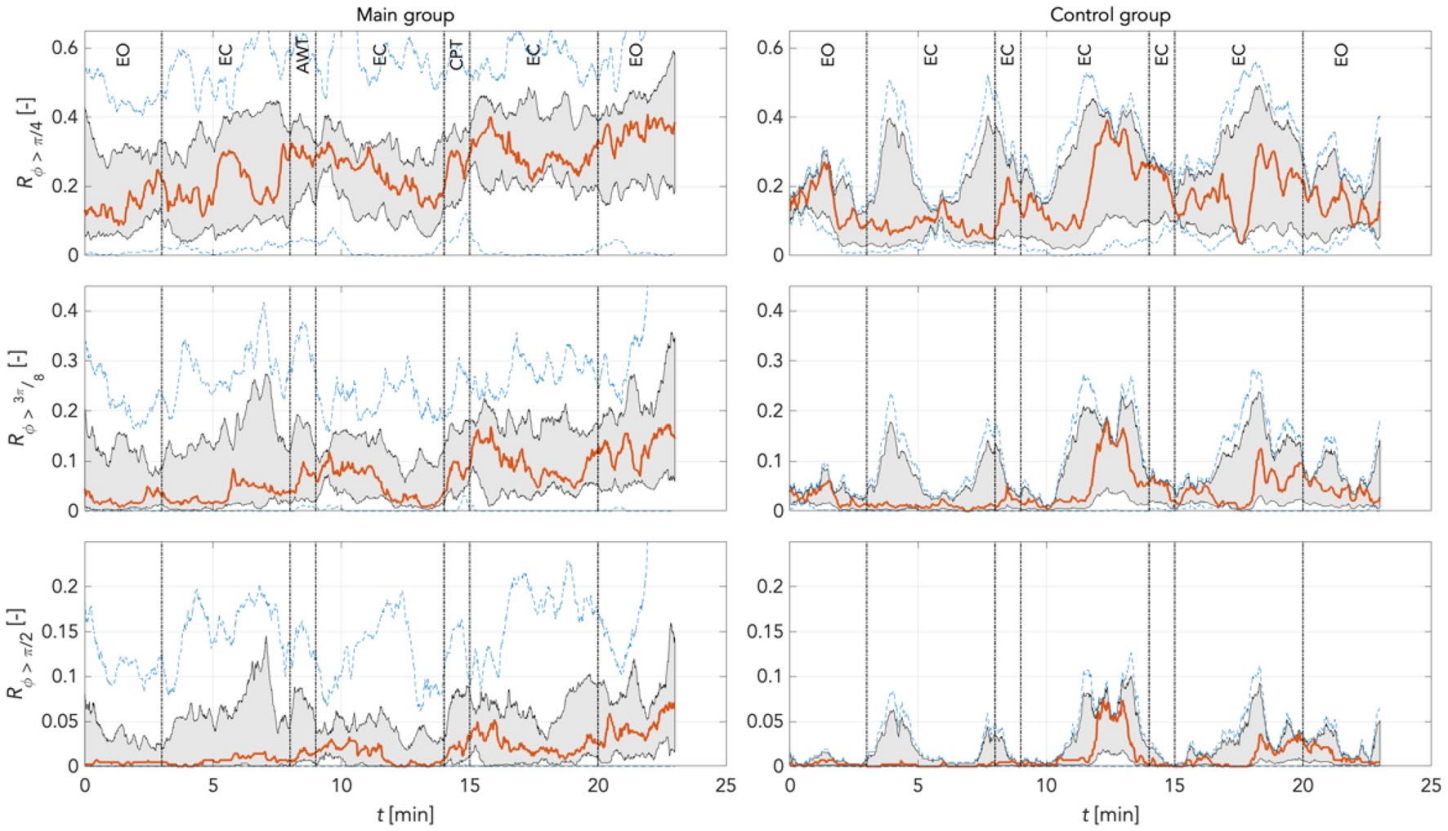
Phase desynchronization for different thresholds for all groups in the LF_Ke band (0.05 Hz – 0.12 Hz) *completely including* 0.1 Hz activity. Widely identical conditions in the main group as in the LF_PF frequency band. Phase desynchronizations in all thresholds for CPT as in LF_Pf frequency band. Stable course during CPT, rapid increases, slow decreases for about 3 min. Here no oscillations of higher frequency and amplitude. Increases of phase desynchronizations with time in all thresholds. In controls oscillations of comparable period duration, yet of different dynamics: steep rising edges followed by slow continuous activity (8 min, 12 min, 17 min). No increases with time in controls. Red: median curve, black vertical lines: stages of the experiment, grey area bounds: 25th and 75th percentiles; blue dashed lines: min and max values.

Figure 16 shows the time courses of the phase desynchronizations for the main and the control groups for frequency boundaries defined as LF_Ke frequency band (0.05 Hz – 0.12 Hz). In the main group, largely identical conditions are found as in the LF_PF frequency band. The response to CPT appears overall at a comparable course as in LF-Pf. During CPT there is a stable course of phase desynchronization in all thresholds, followed by rapid increases, and slow decays for approx. 3 min. Here, however, oscillations of shorter frequency and amplitude are missing. In the control group, spontaneous oscillations of comparable period duration are found, but not only of stronger amplitude, but also of somewhat different dynamics. Here, steep rising edges are found, followed by slow continuous decay of this activity. This is found roughly three times: in the eighth minute, the 12th minute, and in the 17th minute.

### Statistical analyses

For each phase threshold and group (main + random group, control group), 2 (LF and IM band for each frequency definition) × 7 (Repetition) repeated measures ANOVAs were computed. Mauchly’s test indicated that the assumption of sphericity had been violated in the experimental group for $\overline{{R_{\phi > \pi /2} }}$ in Pfurtscheller (χ^2^(20) = 34.3, *p* = 0.03) and Keller frequency band definitions (χ^2^(20) = 32.7, *p* = 0.04) as well as for Keller definition of $\overline{{R_{\phi > \pi /4} }}$(χ^2^(20) = 32.5, *p* = 0.047). Therefore, degrees of freedom were corrected using Greenhouse–Geisser estimates of sphericity (*ε* = 0.48, *ε* = 0.43 and *ε* = 0.41). In the experimental group, the repeated measures ANOVAs for $\overline{{R_{\phi > \pi /4} }}$, $\overline{{R_{\phi > 3\pi /8} }}$, and $\overline{{R_{\phi > \pi /2} }}$, respectively, revealed significant main effects for the factor repetition (all *p* < 0.05; Table 1). This indicates that phase desynchronization values of all relative phase shifts showed a significant increase throughout measurements. The main effects of the factor band as well as the interaction band × repetition did not yield significant results for any frequency band definition and phase shift (all *p* > 0.05; see Table 1).

**Table 1:** Repeated measures ANOVAs in experimental groups (*N* = 13) for different thresholds and frequency band definitions.

Threshold	Parameter	Source	*F*	*P*
$\phi > \pi /4$	*LF_Pf* *IM_Pf*	Repetition	3.57	**0.004**
		Band	0.003	0.96
		Repetition × Band	1.46	0.20
	*LF_Ke* *IM_Ke*	Repetition	3.66	**0.03**
		Band	1.26	0.28
		Repetition × Band	0.91	0.50
$\phi > 3\pi /8$	*LF_Pf* *IM_Pf*	Repetition	4.25	**0.001**
		Band	0.04	0.85
		Repetition × Band	1.39	0.23
	*LF_Ke* *IM_Ke*	Repetition	3.94	**0.002**
		Band	0.83	0.38
		Repetition × Band	0.64	0.70
$\phi > \pi /2$	*LF_Pf* *IM_Pf*	Repetition	4.203	**0.013**
		Band	0.025	0.878
		Repetition × Band	0.81	0.57
	*LF_Ke* *IM_Ke*	Repetition	3.93	**0.022**
		Band	0.39	0.54
		Repetition × Band	0.61	0.73

To further examine the significant main effect of factor repetition in the experimental group, paired t-tests were computed for each frequency band definition and phase threshold. The LF_Ke band showed for $\overline{{R_{\phi > \pi /4} }}$, $\overline{{R_{\phi > 3\pi /8} }}$, and $\overline{{R_{\phi > \pi /2} }}$ a significant increase from stage 1 to 7 after Bonferroni correction (*p* < 0.0125). The LF_Pf band showed a significant increase from stage 1 to 7 for $\overline{{R_{\phi > \pi /4} }}$ and $\overline{{R_{\phi > 3\pi /8} }}$ at *p* < 0.05 and $\overline{R_{\phi > \pi /2}}$ after Bonferroni correction (*p* < 0.0125). Furthermore, the increase from stage 1 to 7 was significant at *p* < 0.05 for $\overline{{R_{\phi > 3\pi /8} }}$, and $\overline{{R_{\phi > \pi /2} }}$. Further paired t-tests showed a significantly higher phase desynchronization during CPT compared to AWT at *p* < 0.05 for LF_Ke at $\phi > 3\pi /8$ and $\phi > \pi /2$ threshold as well as for LF_Pf at $\phi > \pi /2$. This indicates that CPT affected phase desynchronization particularly in the low frequency range.

**Figure 17:**
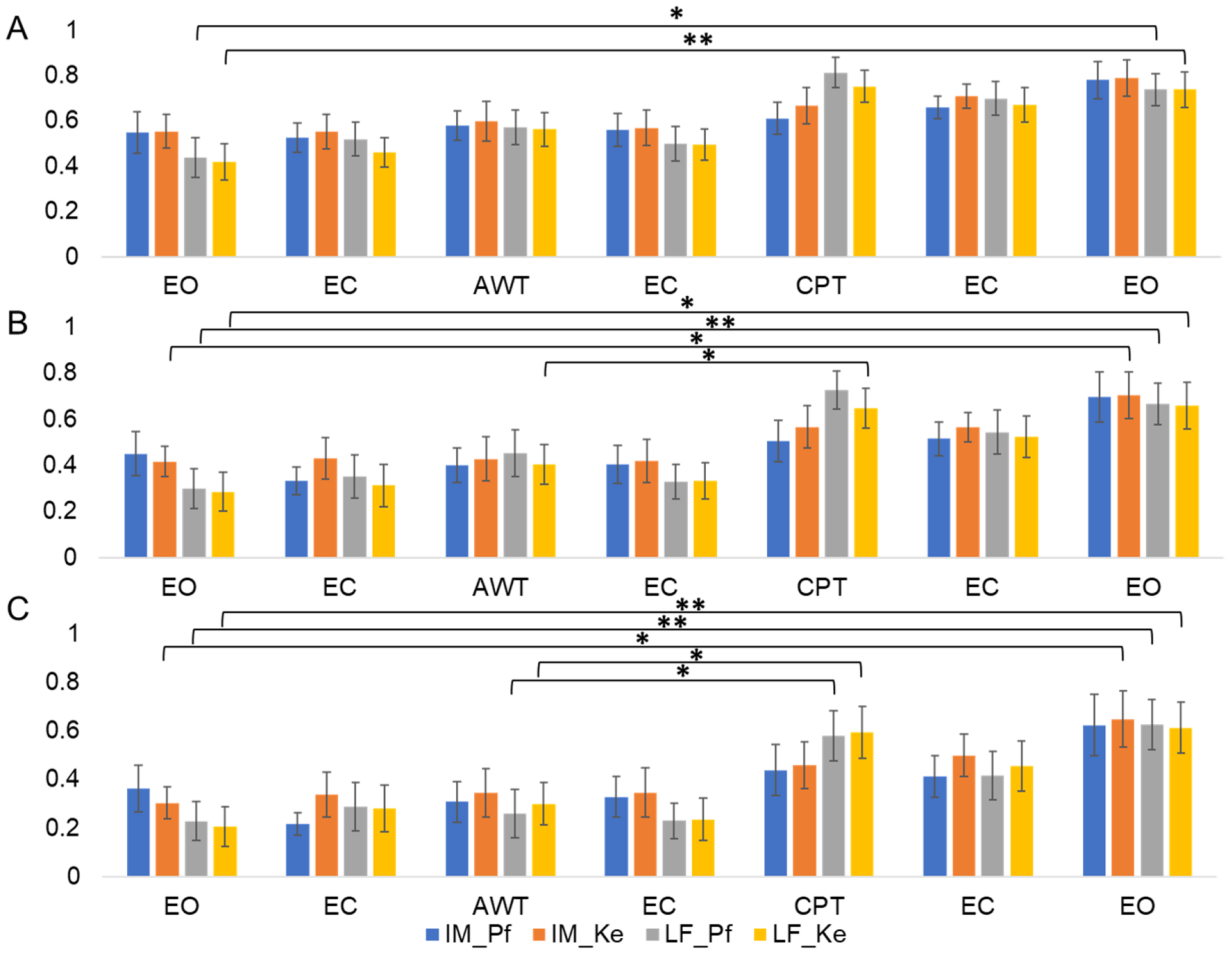
Bar graphs of phase desynchronization for LF and IM frequency band definitions based on Pfurtscheller (Pf) and Keller (Ke), and different thresholds in the main group (*N* = 13). (**A**) Ratio $\overline{R_{\phi > \pi /4}}$ shows a general increasing trend of phase desynchronization and a significant increase from stage 1 to 7 for LF_Pf and LF_Ke. (**B**) Ratio $\overline{R_{\phi > 3\pi /8}}$ showing general increasing trend throughout measurement and significant increase from stage 1 to 7 for IM_Ke, LF_Pf and LF_Ke exhibit significantly higher phase desynchronization during CPT. (**C**) Ratio $\overline{R_{\phi > \pi /2}}$ showing general increasing trend and significant increase from stage 1 to 7 for IM_Ke, LF_Pf and LF_Ke. There is a significant difference between AWT and CPT for LF_PF and LF_Ke. Asterisks indicate (*) *p*-value < .05 or (**) *p*-value significant after Bonferroni correction at *p* < .0125. Error bars show standard error of the mean.

Further 2 (LF and IM band for each frequency definition) × 7 (Repetition) repeated measures ANOVAs were computed for the control group. Neither the main effects of factor repetition and band nor the interaction of repetition × band showed a significant effect for any threshold (all *p* > 0.05; see Table 2). However, descriptively, the IM band definitions displayed an increase of phase desynchronization across stages pointing at a possible time effect (see Fig. 17).

**Table 2:** Repeated measures ANOVAs in control group (*N* = 3) for different thresholds and frequency band definitions.

Threshold	Parameter	Source	*F*	*p*
*ϕ* > *π⁄4*	*LF_Pf* *IM_Pf*	Repetition	0.62	0.71
		Band	1.38	0.36
		Repetition × Band	1.04	0.45
	*LF_Ke* *IM_Ke*	Repetition	0.51	0.79
		Band	0.05	0.85
		Repetition × Band	2.78	0.06
*ϕ* > *3π⁄8*	*LF_Pf* *IM_Pf*	Repetition	0.43	0.85
		Band	1.66	0.33
		Repetition × Band	2.01	0.14
	*LF_Ke* *IM_Ke*	Repetition	1.09	0.42
		Band	2.02	0.29
		Repetition × Band	2.5	0.08
*ϕ* > *π⁄2*	*LF_Pf* *IM_Pf*	Repetition	0.52	0.79
		Band	0.61	0.52
		Repetition × Band	2.59	0.08
	*LF_Ke* *IM_Ke*	Repetition	1.76	0.32
		Band	1.76	0.19
		Repetition × Band	1.00	0.46

**Figure 18:**
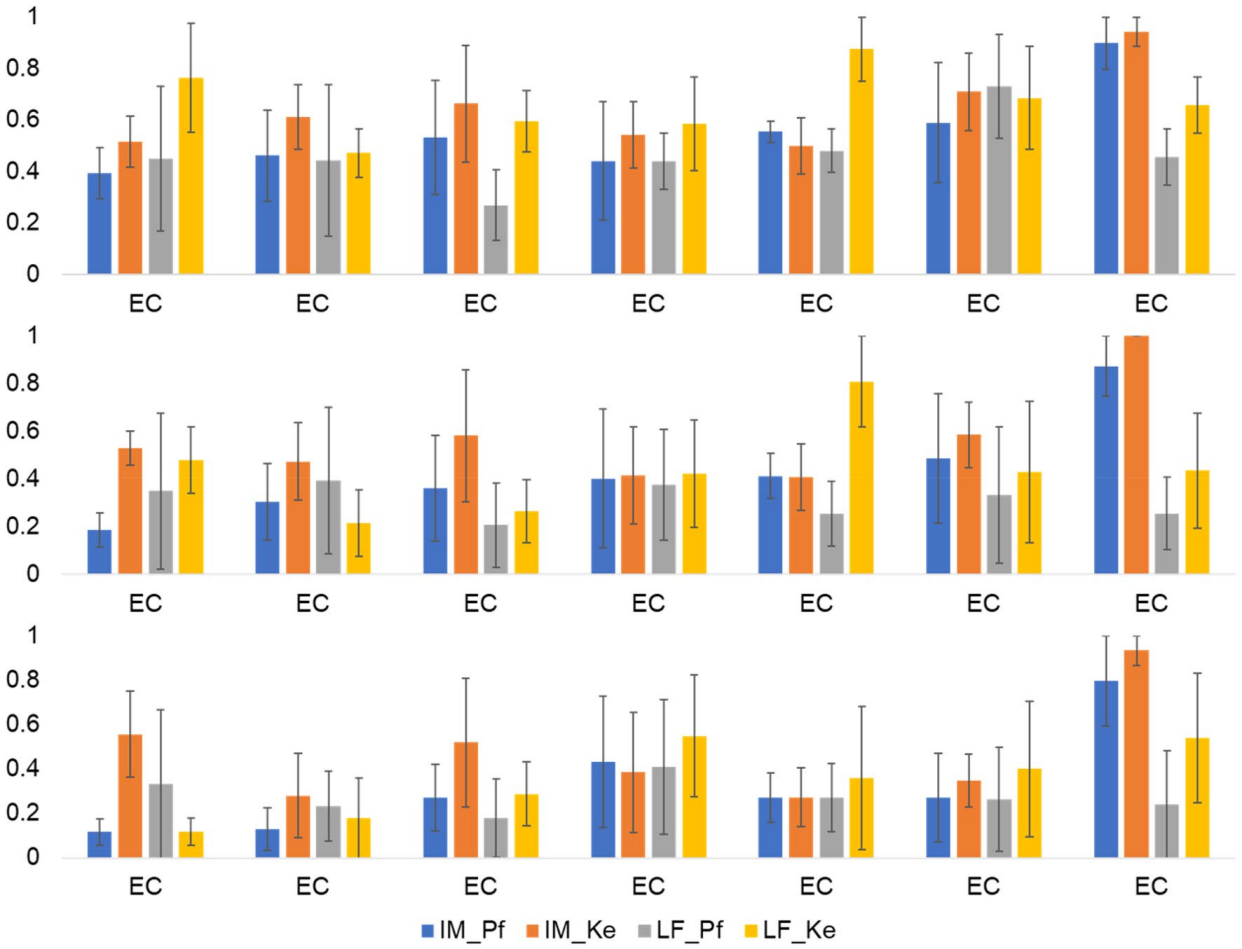
Phase desynchronization for LF and IM frequency band definitions based on Pfurtscheller (Pf) and Keller (Ke), and different thresholds in the control group (*N* = 3). Ratio $\overline{R_{\phi > \pi /4}}$, (**B**) $\overline{R_{\phi > 3\pi /8}}$ and (**C**) $\overline{R_{\phi > \pi /2}}$ shows a general increasing trend of phase desynchronization, however, no significant effects. Error bars show standard error of the mean.

Bar graph analysis underpins these findings. In both LF bands there is a general trend of increasing phase desynchronization over time from stage 1 to 7 in $\overline{R_{\phi > \pi /4}}$. For $\overline{R_{\phi > 3\pi /8}}$ and for $\overline{R_{\phi > \pi /2}}$ this is shown also in IM_Ke. There is a significant difference between AWT and CPT for LF_PF and LF_Ke (Fig. 18).

## Discussion and conclusion

In this study we present an innovative method for the phase shift analysis of cutaneous tissue perfusion that appears promising to assess ANS change processes related to physical or psychological stress. Non-contact mapping of forehead skin perfusion was used to detect spatial phase changes in photoplethysmography imaging (PPGI) data. Thirteen healthy participants were exposed to an ambient/cold pressure water test (AWT, CPT) in randomized order with intermittent resting blocks and compared to three time-control participants who underwent no intervention. Relative phase desynchronization was investigated using three different thresholds ($\phi > \pi /4$, $\phi > 3\pi /8$, and $\phi > \pi /2$). PPGI data was initially filtered using pooled low frequency (LF) and intermediate (IM) frequencies (data not shown). This, however, did not supply sufficient sensitivity for the detection of experimental changes. Following recent evidence showing that LF band activity is better explained by using two subcomponents, data was filtered based on frequency boundaries defined by Pfurtscheller^46^ and Keller^27^. Pfurtscheller et al. suggested to separate LF bands as LFa: 0.05 Hz – 0.1 Hz (referred to by us as LF_Pf), and LFb: 0.1 Hz – 0.15 Hz, whereas Keller et al., used LF_Ke (0.05 Hz – 0.12 Hz) as well as and IM_Ke (0.12 Hz – 0.18 Hz) bands. Thus, Keller LF boundaries included all 0.1 Hz activity reported to exhibit individual variation between 0.075 Hz and 0.12 Hz^26^, which therefore LF_Pf might fall short to reflect. In the same vein, IM_Pf may confound IM activity by including 0.1 Hz activity, which is excluded when starting IM boundaries at 0.12 Hz. Overall, we observed an increase of phase desynchronization throughout the measurement for both LF and IM frequency band definitions in the experimental group and for all phase shift thresholds. A non-significant increase in IM bands of the control group suggests this increase be related to time effects in this frequency range.

CPT compared to AWT significantly enhanced phase desynchronization in both LF bands, though to a larger extent for LF_Ke. Spontaneous increases at seemingly regular intervals unrelated to intervention observed in the control group may indeed indicate that phase desynchronizations were specific for the LF_Pf and LF_Ke bands during CPT. This finding is also of relevance since it supports the notion that the LF_Ke band includes 0.1 Hz activity to a larger extent. Yet, there is ample evidence that LF frequencies reflect activity of different physiological processes. At profound rest, it was shown to contain subharmonics of primary IM band activity^13^. It has been much disputed that conventional LF represents also sympathetic activity^47^. Therefore, measures such as the ratio between LF and HF supposedly indexing autonomic balance have been challenged long^8,48^ and investigating subcomponents of the LF band seems crucial for understanding autonomic dynamics.

Malpas already pointed out that “one must use care in relating changes in the strength of an oscillation in blood pressure and heart rate as definitively due to a change in autonomic control”. But even for ANS control, there are still not all questions sufficiently resolved. Slow oscillations at 0.1 Hz may represent primarily baroreceptor-mediated blood pressure regulation via sympathetic nerve fibres^47^. However, other authors convincingly demonstrated 0.1 Hz oscillations in HRV might rather reflect activity of the unmyelinated vagus nerve originating in the dorsal lateral vagal complex^49^, which was supported by findings that those oscillations were abolished by vagal blockade but only little unaffected by sympathetic blockade^50^. Following other authors who presented a model of frequency-power distribution in skin blood flow^6^, we herewith seem to be able to provide a promising approach to the methodological toolbox needed to improve capturing dynamic ANS regulation on the level of the skin.

According to von Holst^51^, relative coordination is the rule in biological systems, while absolute coordination is the exception. Further, frequency and amplitude responses to various stressors have long been known to exhibit great variation^51^. This has been confirmed more recently in multiple skin locations for IM band frequencies showing great variation with respect to presence of IM band^52^. This has led to the description of these operations as the meshing of cogs and the observed frequencies as clanking of the cogs^53^. Therefore, a more recent study suggested to include ideally a minimum of 2 biological systems in parallel when studying cardiovascular system oscillations^54^.

In general, the coordination of blood flow and intravascular blood volume mandatory for intravascular blood pressure may alter with changes in windkessel properties or with local redistribution of blood flow. This may result in delays reflected as phase differences detected e.g., in cutaneous blood flow. In hard vessels this phase difference may be very small or vanish altogether, whereas soft (dilatated) vessels may exhibit 'large' phase difference. Shifting phases may therefore allow to infer changes in vascular tone. In PPG signals, this may index changes in the tone of pre- and postcapillary sphincters, arterioles or venioles. Given the prominent function of skin temperature regulation, cold pressure test may have had a significant impact.

When combined with established high-resolution algorithms for the analysis of frequencies (e.g., continuous wavelet scalograms), our novel methodology presented here on high resolution of dynamic phase changes should greatly expand and therefore improve the study of rhythmic coordination processes. The improvement comes with transforming the time-domain PPGI signal into an instantaneous phase waveform using the Hilbert transform. Furthermore, the advantage of the non-contact and camera-based approach allows for high spatial resolution of blood volume changes in cutaneous tissues. The matrix of PPGI signals transformed into a matrix of instantaneous phases contained colour coded information on phases relative to the reference point. Determining the number of points of phase shifts on the forehead region exceeding a predefined threshold, allowed evaluation of ANS responses found to be sensitive and capable of determining the state of the subject’s autonomic system.

This is supported by our control group which, however, due to its small sample size should be viewed with caution. The control group showed a clear increase for IM band phase desynchronizations by the end of the experimental session which may be related to the duration of relaxation experienced as tedious since the optimal duration of relaxation should be limited to 7 min^55^. This is reason to assume the present methodology ideally sensitive for the analysis of cutaneous responses qualifying it as a complementary tool for non-invasive diagnosis compared to other evaluation methods, e.g., in the spectral amplitude domain^56,57^, or time-domain analysis^58^.

Our current results rest on analysis of primary cutaneous blood flow rhythmicity. More recently, PPG signals frequently served as an undemanding source for the study of rhythms related to cardiac and respiratory activity (see^59^). On the other hand, methods such as laser Doppler devices proved valuable in physiological studies investigating frequency response characteristics of sympathetically mediated vasomotor activity^60^. Analysis of phase responses are of sound physiological relevance since changes observed in the frequency domain have been burdened by various shortcomings. Among the most important ones are inherent frequency variations or instabilities in biological systems. In systems with low complexity such as medulla fish, inherent frequency variations may amount up to 35% of variation in either direction^51^, and up to 20% in humans reported in osteopathic medicine^61^.

The current findings present a novel technological approach of investigating phase changes in PPGI data. However, reliability of findings should further be investigated in larger samples to differentiate e.g. the long-disputed 0.1 Hz activity in the contexts of LF and IM bands. Furthermore, a variety of physiological factors are known to modulate cardiovascular oscillations. These include the hormonal circuits (HPA axis) regulating blood volume, blood pressure, and vascular tone (renin—angiotensin system; epinephrine and norepinephrine; etc.). Some of these factors can be partly influenced by experimental paradigms, e.g., orthostasis stress (tilt table experiments), physical activity, mental activity (arithmetic tests). Other factors require testing specific patient populations to investigate deviations from physiologic controls. Furthermore, in the present study, we used PPG of the center of the forehead as a reference. In further studies, however, e.g., respiration should be included, since blood flow to the skin and respiration are known to interact intensively^6^. Lastly, physiological changes should be investigated only in a psychometrically validated manner. Essentially, the results of Keller et al. on peripheral 0.15 Hz activity demonstrate the urgent need to interpret physiological measurements with respect to interoceptive processes. This has also been demonstrated by recent results of Pfurtscheller et al. who showed that different levels of fear processing occur at LFa and LFb^26^. Questionnaires such as the Multidimensional Assessment of Interoceptive Awareness^62^ or the state and trait anxiety questionnaire^63^ are important tools for psychophysiological interpretation of phase analyses.

### Outlook

ANS responses at the full scale should be studied further using spatial phase changes as a highly promising method. It will undoubtedly be worthwhile to further investigate these phenomena through a series of additional, comprehensive experiments that could help develop a highly effective tool for evaluating ANS responses. This will eventually help our understanding of the still enigmatic concert of physiological rhythms.
